# Validation of Estimators for Weight-Bearing and Shoulder Joint Loads Using Instrumented Crutches

**DOI:** 10.3390/s23136213

**Published:** 2023-07-07

**Authors:** Marco Ghidelli, Cristina Nuzzi, Francesco Crenna, Matteo Lancini

**Affiliations:** 1 Department of Information Engineering, Università degli Studi di Brescia, 25123 Brescia, Italy; marco.ghidelli@unibs.it; 2 Department of Mechanical and Industrial Engineering, Università degli Studi di Brescia, 25123 Brescia, Italy; cristina.nuzzi@unibs.it; 3 Department of Mechanical, Energy, Management and Transport Engineering, Università degli Studi di Genova, 16145 Genova, Italy; francesco.crenna@unige.it; 4 Department of Medical and Surgical Specialties, Radiological Sciences, and Public Health, Università degli Studi di Brescia, 25121 Brescia, Italy

**Keywords:** partial weight-bearing, instrumented crutches, walking aid, gait analysis, biomechanical model, shoulder joint, shoulder load, load measurement

## Abstract

This research paper aimed to validate two methods for measuring loads during walking with instrumented crutches: one method to estimate partial weight-bearing on the lower limbs and another to estimate shoulder joint reactions. Currently, gait laboratories, instrumented with high-end measurement systems, are used to extract kinematic and kinetic data, but such facilities are expensive and not accessible to all patients. The proposed method uses instrumented crutches to measure ground reaction forces and does not require any motion capture devices or force platforms. The load on the lower limbs is estimated by subtracting the forces measured by the crutches from the subject’s total weight. Since the model does not consider inertia contribution in dynamic conditions, the estimation improves with low walking cadence when walking with the two-point contralateral and the three-point partial weight-bearing patterns considered for the validation tests. The shoulder joint reactions are estimated using linear regression, providing accurate values for the forces but less accurate torque estimates. The crutches data are acquired and processed in real-time, allowing for immediate feedback, and the system can be used outdoors in real-world walking conditions. The validation of this method could lead to better monitoring of partial weight-bearing and shoulder joint reactions, which could improve patient outcomes and reduce complications.

## 1. Introduction

Incremental weight-bearing is often required during post-surgery gait rehabilitation to reduce implant failures and improve soft tissue healing. The Partial Weight-Bearing (PWB) approach includes increasing weight loading on the limb progressively over time, which varies between patients depending on the extent of the injury and the judgment of the clinician [1,2]. Walking aids, such as crutches, can also help reduce instability in patients with after-stroke hemiparesis and are prescribed for weight-bearing on the involved weak lower extremity [3]. During rehabilitation, physiotherapists usually provide instant feedback to the patient regarding posture, load, and step sequence according to his/her perception and experience. The results are thus tied to the therapist’s skills, and the patient’s improvements are evaluated by the therapist’s subjective considerations [4]. Several studies have reported that adequate compliance with lower extremity partial weight bearing is not usually reached, increasing the risk of complications [5,6,7,8]. In [7], none of the patients were able to follow the prescribed reduction of 30% of their body weight (BW), and one-third of them were not aware of their inability to follow the instructions. Another study highlighted that none of the participants managed the prescribed load on the operated limb, especially patients who were 60 years old or more [8]. It is necessary to monitor not only consequences on the affected limb but also secondary symptoms involving the upper extremities, which can be stressed during daily activities if crutches are involved [7,9,10,11]. In [12], shoulder force magnitudes up to 170%BW were measured during crutch-assisted walking with partial or complete unloading of the lower limb. Moreover, the number of repetitions during long-lasting crutch use could lead to shoulder problems as a long-term consequence.

Force platforms and motion capture systems are commonly used to extract kinematics and kinetics information of the gait also when performed with walking aids. Even if gait laboratories could perform highly accurate measurements [13], some limitations must be considered in clinical applications: they are expensive to set up and operate [14]; patients may need to travel to a specialized gait laboratory, and this can be challenging for individuals who have limited mobility; the controlled environment of the laboratory may be a rough simulation of a patient’s real-life walking patterns; and the test takes place over a relatively short period of time [15]. Moreover, the information related to the reaction forces of the shoulders is not usually available in real-time since it requires post-process analysis of the acquired data. 

Recently, several wearable technologies have aimed to bring gait analysis outside laboratory conditions. Many instrumented insoles are used to collect data in daily life activities, and most of them allow real-time acquisition, but they usually require a preliminary calibration to measure forces accurately [14,16,17]. Other research groups and enterprises are improving markerless systems based on inertial measurement units, but they are not yet accurate enough in joint center locations or joint angle estimation [18,19]. Several research groups have created instrumented crutches because they allow the estimation of the patient’s performance during walking and to provide feedback to improve the rehabilitation. The systems developed in [20,21,22,23,24,25,26] measured the force applied to the crutches and their orientation and send the acquired data via wireless communication. For the force measurement, the systems in [21,22] used load cells mounted at the lower end or inside the crutch, while the instrumented crutches in [23,26] used strain gauges to reduce the cost of the device. In [20,21], the acquired data were displayed in real-time on a PC or through a projector to show them to the patient or physiotherapist. In [21,22] vibratory and audio feedback was added to inform the user of overloading or underloading, and in [27], patients showed higher compliance to the rehabilitation goals when audio feedback was provided.

**Figure 1:**
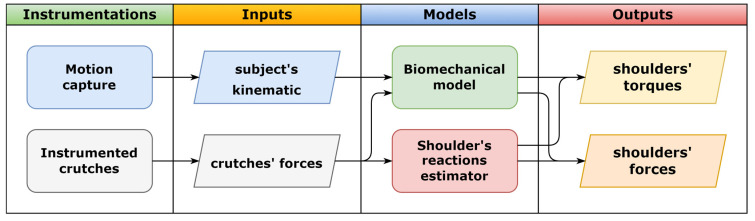
Comparison between the flowcharts of the biomechanical model approach and the shoulder reactions estimator.

In this paper, we provide a preliminary methodological validation of an estimator of the partial weight-bearing of the lower limbs and the shoulder joint’s reaction forces during a walk with instrumented crutches. The estimate was provided by a pair of instrumented crutches to measure the ground reaction forces along their principal axis, and the forces were acquired and processed in real-time, which is suitable to collect data or provide feedback [20,21,22,27,28]. The system is validated on six healthy subjects, and since the estimation of the load on the lower limbs during partial weight-bearing is affected by dynamics factors ignored by the model, the tests were performed varying the walking cadence and the load on the crutches. Two force platforms by BTS were used to collect the reference values of the leg load. The models to estimate the shoulder reactions were identified and tested using the reference values obtained by inverse kinematics using the biomechanical model described in [11] and an optoelectronic motion capture system by VICON. The estimator could produce accurate estimates of shoulder forces, although its torque estimates were less accurate. However, as schematized in Figure 1, it does not require motion capture devices to extract the subject’s kinematics, and the system can also be used outdoors, bringing the analysis to environments outside the laboratory’s conditions. Since neurological conditions, orthopedic problems, and medical conditions could lead to alterations in gait behavior [29], this preliminary validation aimed to promote future investigations to confirm and reinforce the method by expanding the sample size and including impairments.

## 2. Materials and Methods

### 2.1. Experimental Set-Up

The instrumentation used and described in the following chapter is summarized in Table 1, and the main details are listed.

**Figure 2:**
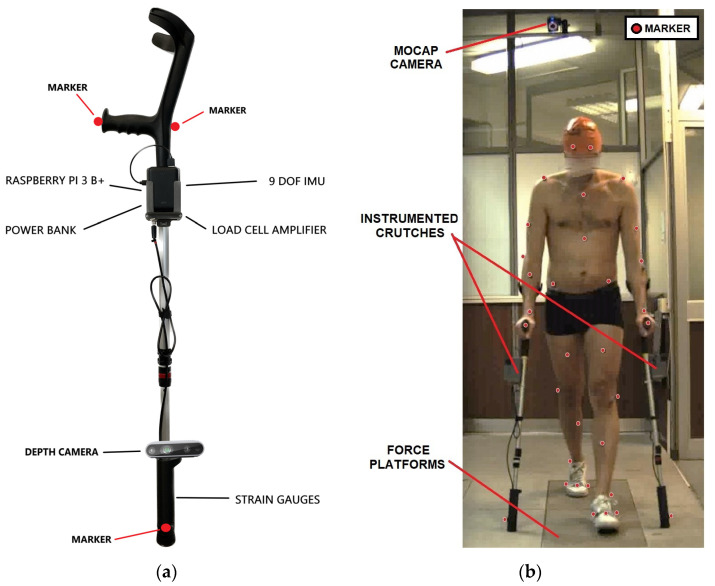
(**a**) Instrumented crutches and marker placement; (**b**) subject walking with instrumented crutches during validation. Red dots highlight the visible markers from the frontal point of view.

The instrumented crutches, shown in Figure 2, measure the force exerted along their principal axis and the crutch’s orientation during assisted walking. They are a new version of the previously developed instrumented crutches [23,24,26] in which the control unit is changed, and they also measure the gait phases using depth cameras mounted on the lower part of the crutches [25,30]. A Raspberry PI 3 B+ controls the data acquisition from the strain gauge bridges fixed close to the tip, and the crutch’s orientation is obtained by an inertial measurement unit. Data are shared wirelessly in an ROS network [31], and a PC under the same network collects them. The force is measured at 60 Hz in a range from 0 to 600 N with 6 N of measurement uncertainty (*p* = 95%) [23]. The total mass of a single crutch, including the acquisition board and the power unit attached close to the handle, is 1 kg.

Besides the instrumented cutches, the experimental set-up included two force plates from BTS Bioengineering to measure each foot’s ground reaction force and a marker-based motion capture system from Vicon Motion Systems with 8 cameras to measure the subject’s kinematics. The sampling frequencies were different for the three systems: the force plates acquired data at 1 kHz, the motion-capture system acquired data at 100 Hz, and the crutches acquired data at 60 Hz, so a resampling procedure was required before starting data processing.

The Vicon Full Body Plug-In Gait model was used for the placement of the markers on the subject’s body [32]. Two additional markers were placed on the first and fifth metatarsus of each foot, and another three markers were fixed on each crutch, as shown in Figure 2. A total of 49 markers were used in each trial. 

A custom-made trigger box was used for data synchronization between the motion capture system, the force platforms, and the instrumented crutches [33]. The internal clock of the trigger box was synchronized with the same NTP server as that used for the instrumented crutches, and when it received a rising edge from the input channel, it published its timestamp in UNIX format through the ROS network. The published timestamp was then associated with the first sample of the motion capture system and the force plates. The crutches were already synchronized with the NTP server, and they saved their timestamp labels in UNIX format.

Vicon’s software Nexus was used to start and stop the acquisition of all the devices involved in the experiment. The analog output channels of the Vicon Lock control box were connected to the BTS’s trigger box and the instrumented crutches’ trigger box. When the Vicon acquisition began, the control box generated a rising edge on the outputs. On the rising edge, the BTS’s trigger box enabled the acquisition from the force plates, and the instrumented crutches’ trigger box sent the start command and its timestamp through the ROS network. The instrumented crutches streamed the data once they received the start command. With a similar approach to the start, the control box generated a falling edge to stop all the acquisitions.

Please note that this configuration was required for validation only; when the instrumented crutches are not used simultaneously with other devices, they can be controlled independently.

### 2.2. Experimental Protocol

The experimental protocol aimed to acquire data useful for validating the proposed PWB and shoulder reaction estimators. Since the effect of the gait dynamics was somehow neglected in the model that determined the PWB from crutches’ forces, the protocol included three different pacing conditions during walking: normal or subject’s preferred rhythm and slow and fast cadence rhythms. These different conditions enabled the verification of the dynamic effects of the proposed approach. A metronome helped the subjects follow a cadence of 50 or 90 steps/min, with the goal of covering reasonable boundaries of a walking cadence with crutches [34,35]. Subjects were asked to walk using the 2-point contralateral pattern [36], and then the tests were repeated with the 3-point partial weight-bearing (3-point PWB) walking pattern [36].

Finally, the protocol required two PWB levels in each condition. To achieve weight-bearing values on the lower limbs comparable with those commonly prescribed by clinicians, the subjects must load 20% or 40% of their body weight on the crutch. In this way, during the 2-point contralateral pattern, the PWB should stay between 60–80%BW, and during the 3-point PWB pattern, it should stay between 20–60%BW. 

In order to verify subject repeatability, at least three trials were conducted for every condition. In addition, before starting the trial sequence, a calibration procedure was necessary to calibrate the motion capture model and properly adjust the force plates to zero. A training session of about 10 min was performed before these calibration procedures to let the subjects familiarize themselves with walking patterns, cadences, and crutch loads.

Table 2 summarizes the protocol, which included a minimum of 33 useful trials for each subject. The crutch loads were checked at the end of each test; if they reached the required value, the trial was accepted, and otherwise, it was repeated. 

The experimental protocol was applied to 6 subjects, 5 male and 1 female with a weight of 78 ± 12 kg, a height of 1.77 ± 0.03 m, and an age of 38 ± 12 years old (mean ± STD). Since at this stage, we were interested in the validation of the proposed approach and not in clinical validation, we performed the tests on healthy subjects only. The study was conducted in accordance with the Declaration of Helsinki, and informed consent was obtained from all subjects involved.

### 2.3. Partial Weight-Bearing Estimation and Validation

Since during assisted walking, the external forces applied to the subject are the ground reaction forces on the feet and on the crutches, the authors believed that the load supported by the user’s lower limbs could be estimated by subtracting the left crutch force (LCF) and the right crutch force (RCF) from the total subject body’s weight (BW), which is the sum of the instrumented crutches’ weight and the user’s weight. The load is then normalized and expressed as a percentage of the total subject’s weight:

(1) $$PWB=\frac{TW-LCF-RCF}{BW}$$

The reference values are obtained from the sum of the left platform force (LPF) and the right platform force (RPF), normalized by the total subject’s weight:

(2) $$\hat{PWB}=\frac{LPF+RPF}{BW}$$

The difference between the two values is considered the error:

(3) $$ERROR=PWB-\hat{PWB}$$

**Figure 3:**
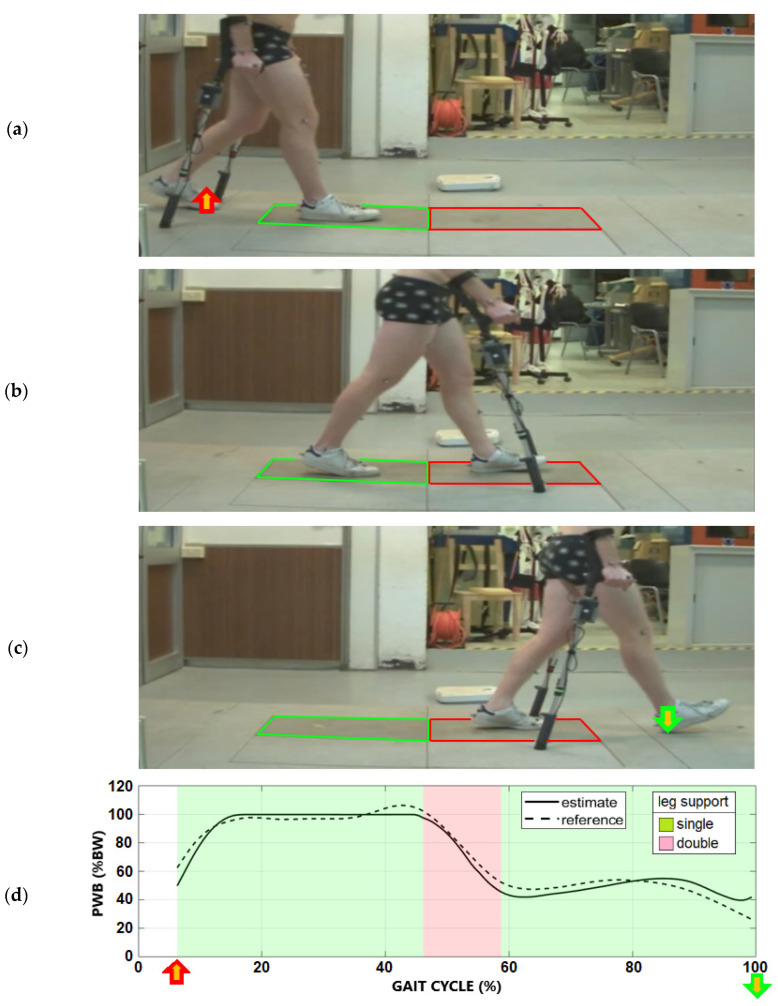
(**a**) The gait event at the start of the interval for the validation of the PWB. The single support phase of the right leg started from this event until the next left foot’s heel contact. (**b**) The double leg support phase with all external forces measured by instrumented crutches and force plates. (**c**) The gait event at the end of the interval for the validation of the PWB. The left leg’s single support phase was between the last toe-off and the next heel contact of the right foot. (**d**) Comparison between the PWB estimated by the instrumented crutches and the reference value from the force plates, shown in the right leg’s gait cycle. The blank background is due to the interval with unknown external forces applied to the foot still resting on the floor.

The error was calculated when all the external forces were known, and the validation intervals were obtained from the gait events recorded by the motion capture. The valid interval started after the last toe-off event outside the force platforms, shown in Figure 3a, and it ended at the first heel contact outside the force platforms, as shown in Figure 3c. The gait cycle was then divided into single and double support; Figure 3b shows the double support phase starting from the first heel contact on the force plate and ending at the next toe-off. Figure 3d shows the PWB estimated by the instrumented crutches compared with the reference during the gait cycle.

Of course, the inertia contribution in dynamic conditions could worsen the results, so a specific testing protocol was proposed to verify this point, varying the user’s walking pace and the crutch loads. The root-mean-square error (RMSE) was used to measure the difference between the values predicted by the estimator and the values observed. The mean error (ME) was used to measure the systematic bias since the RMSE included both stochastic and systematic errors.

### 2.4. Shoulder Reaction Regressions

Shoulder reaction reference values were obtained by the biomechanical model outcomes described in detail in [11]. The model is based on an inverse kinetics analysis of the upper limbs. It was originally created to analyze torques in the sagittal plane, and then it was extended to the other planes, including coupled three-dimensional effects. The inputs required are the subject’s anthropometry, the kinematics of the crutches and the subject’s upper limbs, and the force measured on the crutches. The model considers the hand rigidly connected to the crutch, so the upper limb is described by three rigid segments: the crutch plus the hand, forearm, and arm. The torque and the internal forces are computed at the proximal end of the shoulder or arm. The biomechanical model’s main goal was to obtain an online estimation of the overall shoulder torque; for this reason, a purely mechanical model was considered; this model considers the overall torque required in the articulation to realize the measured movement, and it does not include the forces produced by active muscles on the articulation.

As previously described in the introduction, the purpose of the shoulder loads and torque regression model is to obtain their estimates without measuring upper limb kinematics. In such a way, the biomechanical model is not required, and an online, rough indication of shoulder loads and torque can be directly given to the patient and to the physician. Since the biomechanical model does not need the ground reaction forces on the feet, any gait cycle is valid to create the regression dataset to compute the shoulder values.

The wide dataset was then divided into identification and validation datasets. For this separation, we considered that, in accordance with the test protocol, each condition was repeated at least three times, so one trial was randomly assigned to the validation dataset, while the remaining trials were assigned to the identification dataset. With this procedure, we maintain a balanced representation of the population and conditions between the datasets with good numerosity: 2/3 for the identification and 1/3 for the validation. The model estimated the peaks and the root mean square (RMS) of the shoulder torques and forces, calculated by the biomechanical model in every stance phase of the crutches. Each subject performed at least two stance phases of the crutch on both sides in every trial.

Since the purpose is to give an online indication to the patient, the time histories of the loads at the shoulders are not required, while peak and root mean square values are certainly more informative. The corresponding values were computed for each crutch cycle and the force is normalized by the subject’s weight while the torque is normalized by the subject’s weight times the subject’s height. 

In the first regression attempt, we considered forces in the sagittal plane (anterior–posterior and vertical directions) and the torque exerted along an axis perpendicular to this plane (mediolateral). In fact, these directions were the most significant in a previous study [11], as they include the force in the vertical direction and the torque needed to maneuver the crutches during the gait cycle. To simplify the model and focus only on relevant predictors, we investigated the correlation between possible influencing factors and shoulders forces and torques before proceeding with the regression. We considered the overall set of available parameters, including the most evident crutch force, anthropometric parameters, kinematic parameters, stance time, and walking pattern. Then, we included all possible couple interactions. For both shoulder forces and torques, we classified possible predictors in decreasing order of correlation with a clear discrimination at a certain correlation level, enabling a clear selection of the most significative parameters for our purposes.

## 3. Results

### 3.1. Partial Weight-Bearing

Table 3 and Table 4 report the analysis of variance (ANOVA) results of the RMSE and the ME respectively. The walking pattern was not statistically significant for the RMSE and the ME; however, all the other parameters (cadence, crutch load, subject, and support status) were statistically significant in both the PWB’s RMSE and ME.

In Table 3, the cadence has the highest F value, showing a higher influence on the RMSE; by contrast, in Table 4 the ME is more affected by the support status of the legs.

**Figure 4:**
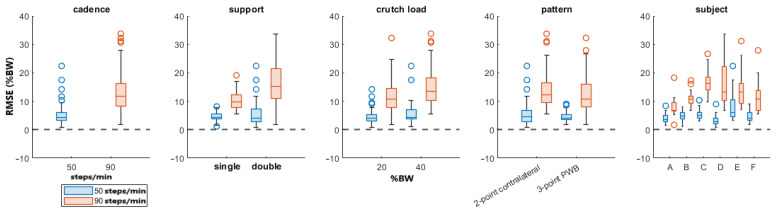
Boxplots of the PWB’s RMSE. The line inside each box is the sample median, and the top and bottom edges of each box are the upper and lower quartiles (0.75 and 0.25), respectively. The distance between the top and bottom edges is the interquartile range.

Figure 4 shows the boxplots of the RMSE for all the parameters divided by the cadence since it has the highest F-value.

**Figure 5:**
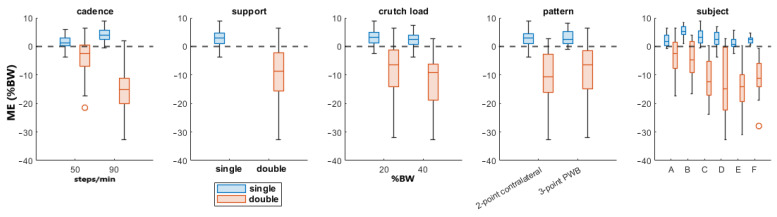
Boxplots of the PWB’s ME. The line inside of each box is the sample median, and the top and bottom edges of each box are the upper and lower quartiles (0.75 and 0.25), respectively. The distance between the top and bottom edges is the interquartile range.

Figure 5 shows the boxplots of the ME for all the parameters divided by the gait support since it had the highest F-value in Table 4.

**Figure 6:**
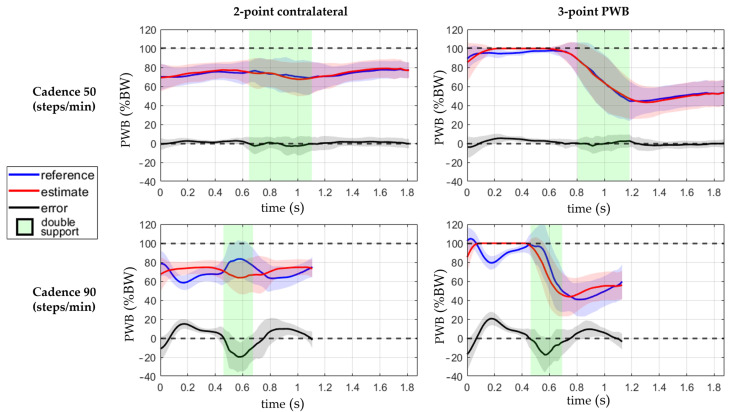
Comparison between the estimated PWB and the reference and the status of the leg support. The line represents the mean value, and the standard deviation is shown with the colored band around the mean. The data are from four conditions combining the walking pattern while walking at 50 or 90 steps/min. Data are visualized with respect to time, and the green and red backgrounds indicate the double-leg support interval.

**Figure 7:**
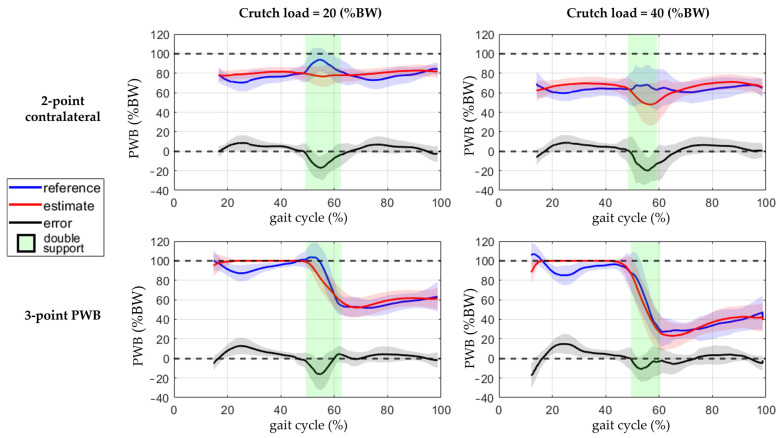
Comparison between the estimated PWB and the reference during the gait cycle and the status of the leg support. The line represents the mean value, and the standard deviation is shown with the colored band around the mean. The data are from four conditions combining the walking pattern while loading 20 or 40% of the body weight on the crutch. Data are visualized with respect to the percentage of the gait cycle, and the green and red backgrounds indicate the gait cycle interval with double-leg support. The missing data between 0–15% of the gait cycle are due to the interval with unknown external forces applied to the foot still resting on the floor.

The PWB’s reference, estimate, and error are shown with respect to the time in Figure 6 and with respect to the gait cycle in Figure 7. The data are compared between the gait pattern and the cadence in Figure 6 and the crutch load in Figure 7; moreover, the double-leg support is shown in the background.

### 3.2. Shoulder Joint Reactions

According to the procedure described in Section 2.3, we ordered the possible predictor, including their interaction, according to their Pearson correlation coefficient with shoulder forces and torque, and we obtained a clear separation for parameters above 0.65 for the torque and 0.90 for the force. We considered a regression model including such parameters and their interactions and with the possibility to consider a second-order model. The parameters above the Pearson correlation index thresholds (0.65 for torques and 0.90 for forces) are shown in Table 5.

Moreover, in order to maintain a clear physical correspondence of the model, we considered the same predictors set for both force and torque, so we assume that this parameter set is significant for the general shoulder load.

**Figure 8:**
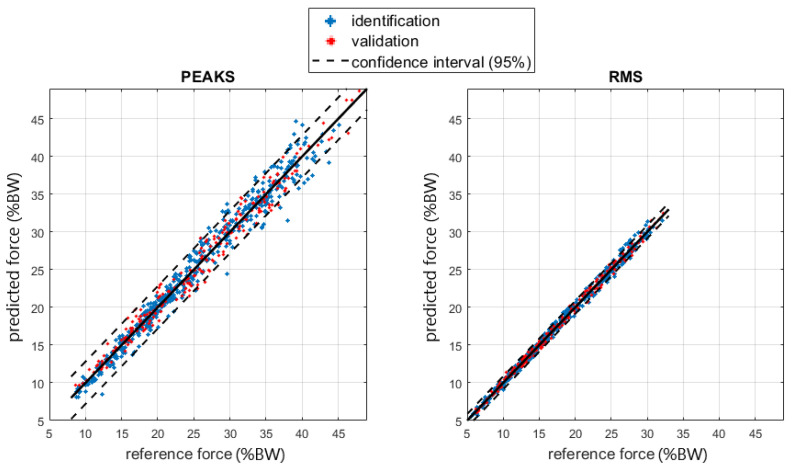
Regression of the shoulder joint force RMS and peaks. The force is shown as a percentage of the subject’s BW.

**Figure 9:**
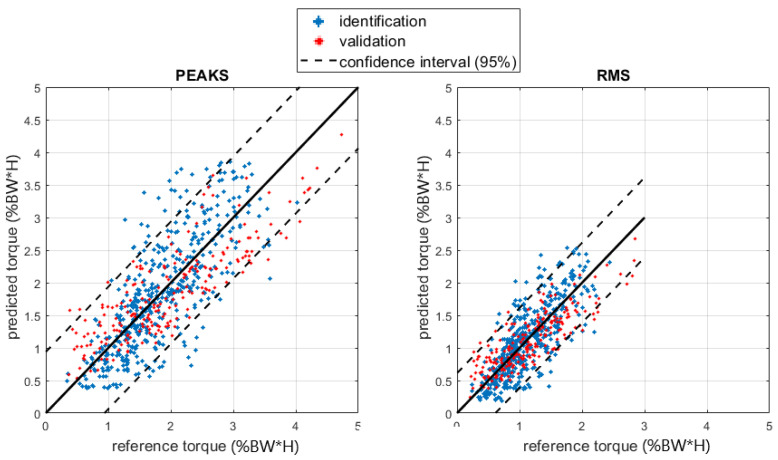
Regression of the shoulder joint torque. The torque is shown as a percentage of the BW times the height (H) of the subject.

Figure 8 and Figure 9 show the regressions of the shoulder joint force and torque.

Table 6 and Table 7 display the RMSE values for the shoulder joint force and torque.

## 4. Discussion

The presented approach estimated the partial weight-bearing from the measurement of a pair of instrumented crutches. The ANOVA results of Table 3 and Table 4 show that the error was strongly influenced by the cadence and the legs support. A higher walking pace produced a higher inertia contribution, and the double-leg support also included the toe-off and heel contact events, which generated higher accelerations than the midstance phases. This confirmed that higher dynamic conditions worsen the PWB estimation. When the cadence was about 90 steps/min, the RMSEs reached more than twice the value reached walking at 50 steps/min in all the parameter combinations, as shown in Figure 4. Moreover, the double-support condition was more affected by a high systematic error than the single support, as highlighted by the boxplots in Figure 5. The highest RMSE value is observed during double-support walking at a speed of 90 steps/min, and this can be explained by the contribution of the mean error in this combination, indicating a systematic bias in the measurement. This means that real-time values are more reliable with lower cadence and speed, as could happen during the first rehabilitation days. Typical values of cadence for assisted walking with crutches are 73.2 ± 8.5 steps/min [34,35].

Moreover, if the gait phases are detected, both cadence and gait support are known, and the PWB estimation can be corrected by removing the systematic error. There are several devices that can be used to assess gait phases, but they may have limitations. IMUs or instrumented insoles require the patient to wear the device and sometimes require calibration. Fixed RGB or infrared cameras must be installed in some motion capture systems, which limits their use to specific locations. These devices also require a post-processing stage sometimes and cannot provide real-time feedback. While our instrumented crutches may provide gait phase detection, the process still needs to improve the reliability and elaborate data in real-time [25,30].

**Figure 10:**
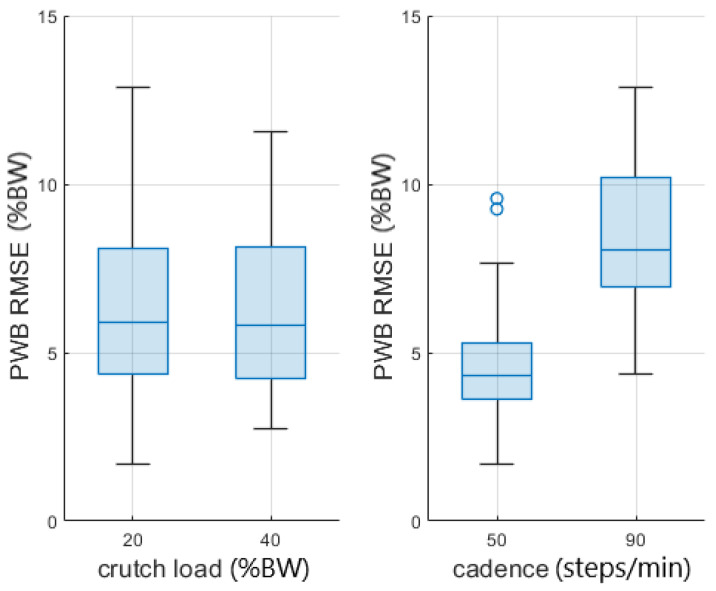
PWB’s RMSE boxplots in the single-support phase of the affected limb. The line inside of each box is the sample median, and the top and bottom edges of each box are the upper and lower quartiles (0.75 and 0.25), respectively. The distance between the top and bottom edges is the interquartile range.

In the three-point PWB gait pattern, the PWB estimation during the single support of the unaffected limb was not useful for rehabilitation purposes, and the crutches were lifted from the ground most of the time with saturation at 100%BW of the prediction. As shown in Figure 6 and Figure 7, the unaffected limb single-support was in the interval before the double support for the three-point PWB pattern, and the errors reached the highest positive values. If only the affected limb single-support interval was considered during three-point PWB walking in all the other conditions, the RMSE was about 6.8%BW and the ME was 1.3%BW, and as shown by the boxplots in Figure 10, the RMSE was not influenced by the crutch load but only by the cadence.

**Figure 11:**
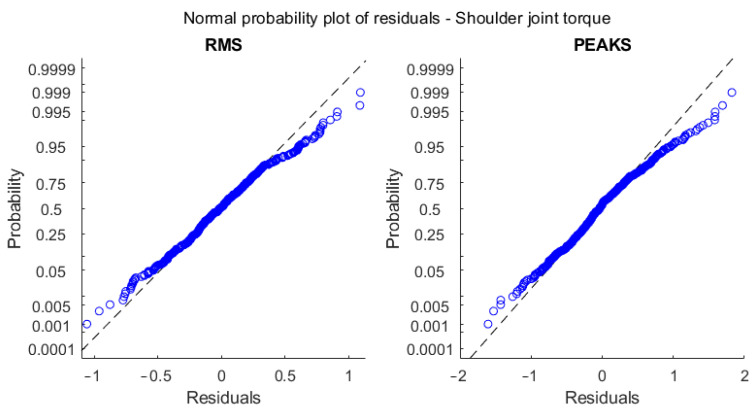
Normal probability plots of regression residuals of the identification subset for RMS and peak shoulder force.

The shoulder regression model was based on RMS and the peak values for crutch force and their interaction with the subject’s height and squared height. The regression was good regarding shoulder force (R^2^ about 1), while it was more critical for torques (R^2^ about 0.6). The residuals for the RMS and peak force regression models followed a normal distribution, as shown in the normal probability plots in Figure 11, so the random behavior of the residuals was confirmed.

**Figure 12:**
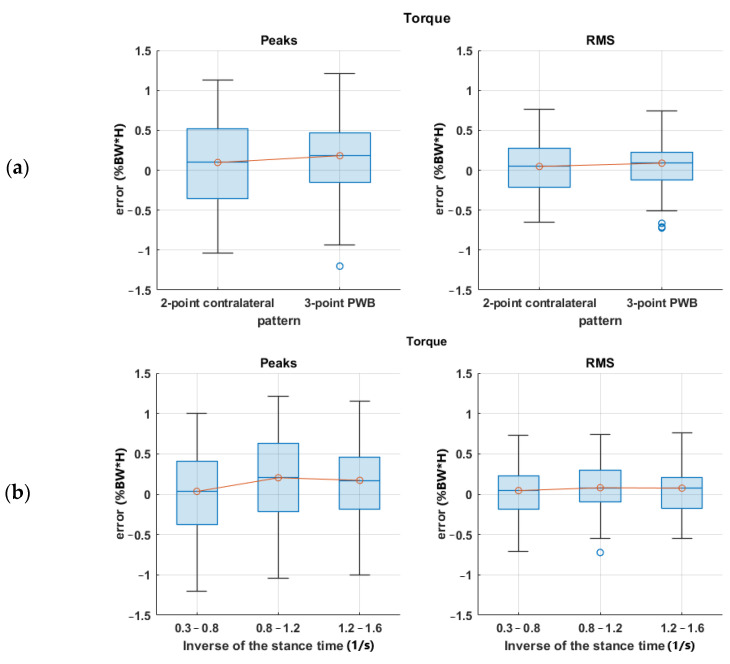

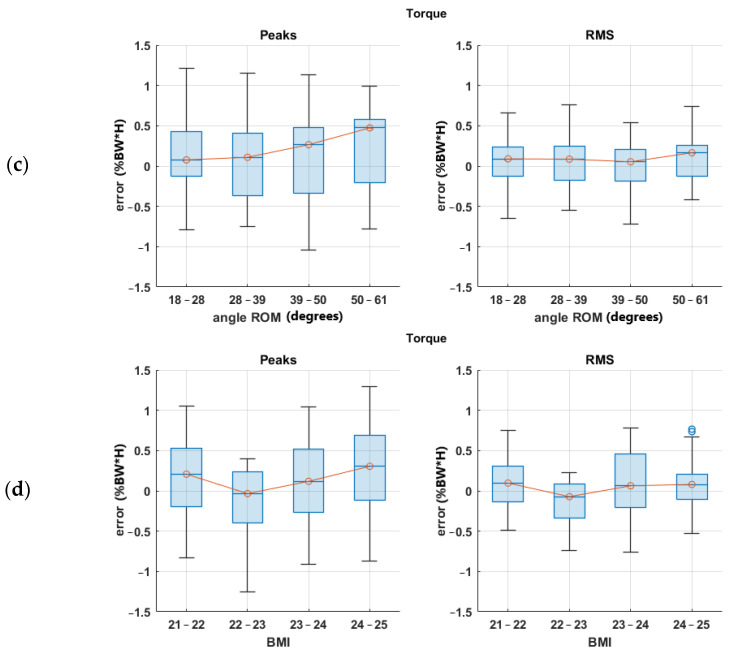
Shoulder torque RMSE boxplots for (**a**) walking pattern; (**b**) the inverse of stance time; (**c**) crutch angle range of motion; (**d**) BMI.

Regarding torque, the R^2^ was lower, and residuals were less normal. A possible justification relies on the equations describing the inverse dynamics: they are based on some anthropometric parameters determined with the help of statistical tables based on specific population samples. Precise evaluation of the effect of anthropometric parameters uncertainties on the inverse dynamics results uncertainty is complex due to non-linearities in the model equations, the large number of possible influence factors, and the recursive relation that moves the results from a distal to a proximal element. A previous investigation [37] applying the simplification principles described in [38] evidenced that anthropometric parameters may heavily affect the inverse kinetics analysis and, in our case, the reference shoulder torque and force. In the force case, equations included only segment masses and their center of mass acceleration. Torque computation also requires the segment’s inertial properties, which generally suffer from a larger uncertainty. Moreover, the correlation analysis excluded anthropometric parameters such as mass and height from the regression due to their poor correlation. The prediction error for torque as a function of the BMI anthropometric parameter can be checked with the box plots in Figure 12, in which no explicit dependency is shown, so the model validity was confirmed, and the reason for the lower torque prediction performance is not clear yet. Of course, a more complex regression model including a larger set of factors improves the correlation at the expense of introducing a large set of quantities in the description. Since our intention here was to give a rough but immediate and easily obtainable estimation to the patient and the clinician to improve gait management, we prioritized model simplicity. To this end, the regression proposed seems to be sufficiently accurate. In fact, shoulder forces were estimated with an RMS error of about 1.5% of body weight, while for torques, the RMS error was about 0.5% of the product body weight times height on a scale ranging up to 4.0 %BW*H, i.e., about 12% of the full scale of the values recorded. Such values are sufficient for a rough indication to the end user and the therapist.

## 5. Conclusions

The presented approach estimated the PWB using instrumented crutches, and the results showed that the error was strongly influenced by walking cadence and leg support. Higher walking speeds and double-leg support result in higher errors in the PWB estimation. The real-time PWB values were more reliable with lower cadence and speed, which were typical values for assisted walking with crutches, and the RMSE was not influenced by crutch load. In the three-point PWB gait pattern, PWB estimation during the unaffected limb’s single support was saturated at 100%BW, but this is not useful for rehabilitation purposes.

The study also developed a regression model for estimating shoulder force and torque during crutch-assisted gait. The model is based on RMS and peak values of crutch force and considers the subject’s height and squared height. The regression model provided good estimates for shoulder force (R^2^ about 1) but was less accurate for torque (R^2^ about 0.6). The less normal distribution of the torque residuals was possibly due to uncertainties in the anthropometric parameters used in the inverse dynamics equations, and a more complex regression model would improve the correlation.

The main goal was to conduct a preliminary methodological study to validate the load estimator applied on the legs using instrumented crutches for force measurement. The inclusion of a larger sample size and a more balanced gender distribution could have provided more generalizable results. Nevertheless, this preliminary study provided promising results that demonstrate the validity and effectiveness of the load estimator system. In the future, studies should include a larger number of participants and adequate gender representation to confirm and strengthen the preliminary findings. Additionally, future studies should consider testing the system under different walking conditions, such as different ranges of speeds, inclines, and terrain types to further validate its accuracy and effectiveness. From the results obtained, it can be assumed that cadence has more effects on the PWB estimation, and it should be investigated more deeply, while the crutch load could be self-selected by the subject or indicated by the therapist since it has less influence. Furthermore, the estimator should also be checked with different impairments since the deviations in the gait could alter the predictions.

## Figures and Tables

**Table 1:** Summary of instrumentations and methods in the experimental setup.

Instrumentation	Details
Instrumented crutches	Range: 0–600 N Uncertainty: 6 N (*p* = 95%) Crutch mass: 1 kg Sampling frequency: 60 Hz Data sharing protocol: ROS Time synchronization: NTP server
Optoelectronic motion capture system (Vicon Motion Systems Ltd., Yarnton, UK)	Cameras: 8 Volume: 6 × 3 × 3 m Marker protocol: ○ full body VICON plug-in gait (39 markers) ○ 2 markers on each foot ○ 3 markers on each crutch Sampling frequency: 100 Hz
LockLab Control Box (Vicon Motion Systems Ltd., Yarnton, UK)	Time synchronization: generates trigger output
BTS force platforms (BTS S.p.A., Garbagnate Milanese, Italy)	Dimensions: 0.8 × 0.6 m Range: 2 kN Uncertainty: Sampling frequency: 1 kHz Time synchronization: receives start/stop trigger from LockLab
Crutches Trigger box	Available I/O channels: 3 Data sharing protocol: ROS Crutch time synchronization: NTP server System time synchronization receives a start/stop trigger from LockLab

**Table 2:** List of conditions of the experimental protocol.

Min. Number of Valid Tests	Conditions
1×	Static test on the first force plate
1×	Static test on the second force plate
1×	Vicon functional calibration
3×	Normal gait, self-selected speed, no crutches
3×	Normal gait, slow (50 steps/min), no crutches
3×	Normal gait, fast (90 steps/min), no crutches
3×	2-point contralateral, slow (50 steps/min), 20%BW
3×	2-point contralateral, slow (50 steps/min), 40%BW
3×	2-point contralateral, fast (90 steps/min), 20%BW
3×	2-point contralateral, fast (90 steps/min), 40%BW
3×	3-point PWB, slow (50 steps/min), 20%BW
3×	3-point PWB, slow (50 steps/min), 40%BW
3×	3-point PWB, fast (90 steps/min), 20%BW
3×	3-point PWB, fast (90 steps/min), 40%BW

**Table 3:** ANOVA of the PWB’s RMSE.

Parameter	Sum of Squares	Degrees of Freedom	Mean Squares	F	*p*-Value
Cadence	4242	1	4242	264	<0.05
Crutch load	345	1	345	21	<0.05
Pattern	13	1	13	1	0.36
Subject	860	5	172	11	<0.05
Support	811	1	811	50	<0.05
**Error**	4113	256	16		
**Total**	10,624	265			

**Table 4:** ANOVA of the PWB’s ME.

Parameter	Sum of Squares	Degrees of Freedom	Mean Squares	F	*p*-Value
Cadence	1346	1	1346	46	<0.05
Crutch load	361	1	361	12	<0.05
Pattern	37	1	37	1	0.26
Subject	1336	5	267	9	<0.05
Support	10,177	1	10,177	348	<0.05
**Error**	7471	256	29		
**Total**	20,961	265			

**Table 5:** Pearson correlation coefficients between the shoulder load and torque and some of the parameters investigated. The * (multiplication) operator yields the product of its operands.

Parameters	Correlation with Shoulder Vertical Force		Correlation with Shoulder Mediolateral Torque	
	RMS	Peaks	RMS	Peaks
Crutch force RMS	1.00	0.96	0.74	0.74
Crutch force RMS * height	1.00	0.96	0.73	0.73
Crutch force RMS * height^2^	0.99	0.95	0.71	0.71
Crutch force peak	0.93	0.98	0.68	0.74
Crutch force peak * height	0.93	0.98	0.67	0.73
Crutch force peak * height^2^	0.93	0.98	0.66	0.71
Crutch force RMS * BMI	0.86	0.86	0.54	0.56
Crutch force RMS * body mass	0.86	0.86	0.52	0.54
Crutch force peak * BMI	0.80	0.87	0.49	0.56
…	…	…	…	…

**Table 6:** RMSE and R^2^ values of the shoulder joint force.

Shoulder Joint Force	RMSE (%BW)	R^2^	Dataset
RMS	0.45	1.00	Identification
	0.42	1.00	Validation
Peak	1.5	0.97	Identification
	1.4	0.98	Validation

**Table 7:** RMSE and R^2^ values of the shoulder joint torque.

Shoulder Joint Torque	RMSE (%BW*H)	R^2^	Dataset
RMS	0.34	0.61	Identification
	0.32	0.68	Validation
Peak	0.55	0.61	Identification
	0.52	0.69	Validation

## Data Availability

Data available on request due to privacy restrictions.
